# Hair‐Like Flexible Airflow Sensor for Large‐Area Airflow Sensing

**DOI:** 10.1002/advs.202510741

**Published:** 2025-09-09

**Authors:** Yingxi Xie, Feilong Liu, Yongchao Luo, Na Lin, Xiaohua Wu, Zeji Wu, Longsheng Lu

**Affiliations:** ^1^ School of Mechanical & Automotive Engineering South China University of Technology Guangzhou 510641 China; ^2^ School of Electronic and Information Engineering Guangzhou City University of Technology Guangzhou 510800 China; ^3^ National Science and Technology Venture Capital Development Center Beijing 100036 China

**Keywords:** flexible airflow sensors, fast response time, hair‐like structures, large‐area airflow sensing, wide detection range

## Abstract

Recently, flexible airflow sensors have attracted significant attention due to their impressive characteristics and capabilities for airflow sensing. However, the development of high‐performance flexible airflow sensors capable of sensing airflow over large areas remains a challenge. In this work, it is proposed that a hair‐like flexible airflow sensor, based on laser direct writing and electrostatic flocking, offers an efficient technology for airflow sensing. The airflow sensor exhibits high sensitivity (5.25% s m^−1^), fast response time (39.83 ms), a wide detection range (3.48 – 18.36 m s^−1^), minimal disturbance to the airflow field, simplified fabrication, and cost efficiency. The airflow sensor, with excellent conformal monitoring capabilities, is capable to detect airflow on different surfaces. The sensor's excellent flexibility and efficient fabrication process enable it to be easily integrated into arrays and deployed on large‐area surfaces to provide information, including airflow velocity, direction, and point of incidence. The hair‐like airflow sensor has significant potential for applications in environmental monitoring, intelligent robots, wearable electronics, and tactile sensing.

## Graphical abstract

A hair‐like flexible airflow sensor is designed for large‐area airflow sensing with carbon fibers implanted on a laser‐induced graphene substrate. The flexible airflow sensor features a wide detection range and fast response time, effectively identifying airflow changes. It is expected to demonstrate reliability in large‐area airflow sensing in numerous applications through its excellent performance.

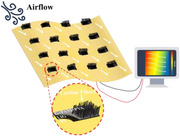

## 1 Introduction

With the development of fields such as aerospace, environmental monitoring,^[^ ^1^ ^]^ wearable devices,^[^ ^2^ ^]^ and intelligent robots, the ability to sense airflow over large areas has become increasingly important in flight perception,^[^ ^3^, ^4^ ^]^ monitoring of minor environmental disturbances,^[^ ^5^ ^]^ and tactile sensing.^[^ ^6^, ^7^ ^]^ Large‐area airflow sensing typically refers to sensing key airflow parameters, such as velocity and direction, over areas larger than several square centimeters through distributed sensor arrays. Airflow sensors, as key components of large‐area airflow sensing systems, have attracted extensive attention. However, traditional airflow sensors have problems such as rigid structure, low sensitivity, and slow response time, which seriously restrict their application. Therefore, flexible airflow sensors that can conform to curved surfaces for large‐area airflow sensing are highly desirable for the development of intelligent aircraft skins, electronic skins, and embodied intelligence. Airflow sensors are generally divided into piezoresistive,^[^ ^8^, ^9^, ^10^ ^]^ piezoelectric,^[^ ^11^ ^]^ optical,^[^ ^12^, ^13^, ^14^ ^]^ magnetoelectricity,^[^ ^15^ ^]^ triboelectric‐nanogenerators‐based,^[^ ^16^ ^]^ and thermal types.^[^ ^17^, ^18^, ^19^ ^]^ Among them, piezoresistive airflow sensors are widely employed due to their stability and simple structure.^[^ ^20^ ^]^

The performance of piezoresistive airflow sensors can be effectively improved through unique structural designs,^[^ ^21^, ^22^, ^23^, ^24^ ^]^ such as thinmembranes, network structures, single fibers, and fluffy structures. Huang et al.^[^ ^25^ ^]^ developed an airflow sensor based on a single ultra‐long silicon nanowire that can effectively monitor wind speed, incidence position, and incidence angle. Inspired by bat wing membranes, Zhou et al.^[^ ^26^ ^]^ developed an airflow sensor based on graphene/single‐walled nanotube‐Ecoflex membranes that can effectively distinguish airflow intensity and spatial distribution. Jiang et al.^[^ ^27^ ^]^ developed a high‐performance airflow sensor based on suspended carbon nanotube networks that established a fast response time and high sensitivity. Wang et al.^[^ ^28^ ^]^ proposed ultrasensitive all‐textile airflow sensor based on carbonized silk fabric (CSF) with in situ grown fluff‐like carbon nano‐tubes (CNTs) (CNTs/CSF) by simulating spider fluffy hair, which can effectively monitor the increase and decrease of airflow velocity and provide danger warnings for the blind. However, the development of piezoresistive airflow sensors remains inadequate and still faces several critical challenges, such as complex and costly fabrication processes, significant interference with the original airflow field, and unsatisfactory performance, all of which restrict their practical application. Therefore, the efficient fabrication and rational design of sensitive structures of airflow sensors are urgently needed to improve the performance and meet the demands of airflow sensing on large‐area surfaces.

Herein, inspired by human hair, a high‐performance and reliable hair‐like flexible airflow sensor, fabricated using laser direct writing and electrostatic flocking, is proposed. This airflow sensor is one of the best performing hair‐structure airflow sensors, exhibiting high sensitivity (5.25% s m^−1^), fast response time (39.83 ms), wide detection range (3.48 – 18.36 m s^−1^), and minimal disturbance to the airflow field. In this work, the airflow sensor is integrated in to arrays and capable to detect global and local variations in airflow. To demonstrate its versatility, the sensor array is integrated into a glove to develop a smart glove, showcasing its capability for distributed airflow sensing. With its excellent performance, this airflow sensor array enables reliable sensing of airflow changes at various locations within a large area. It has significant potential for application in environmental monitoring, tactile sensing, embodied intelligence, and wearable electronics.

## 2 Results and Discussion

**Figure 1:**
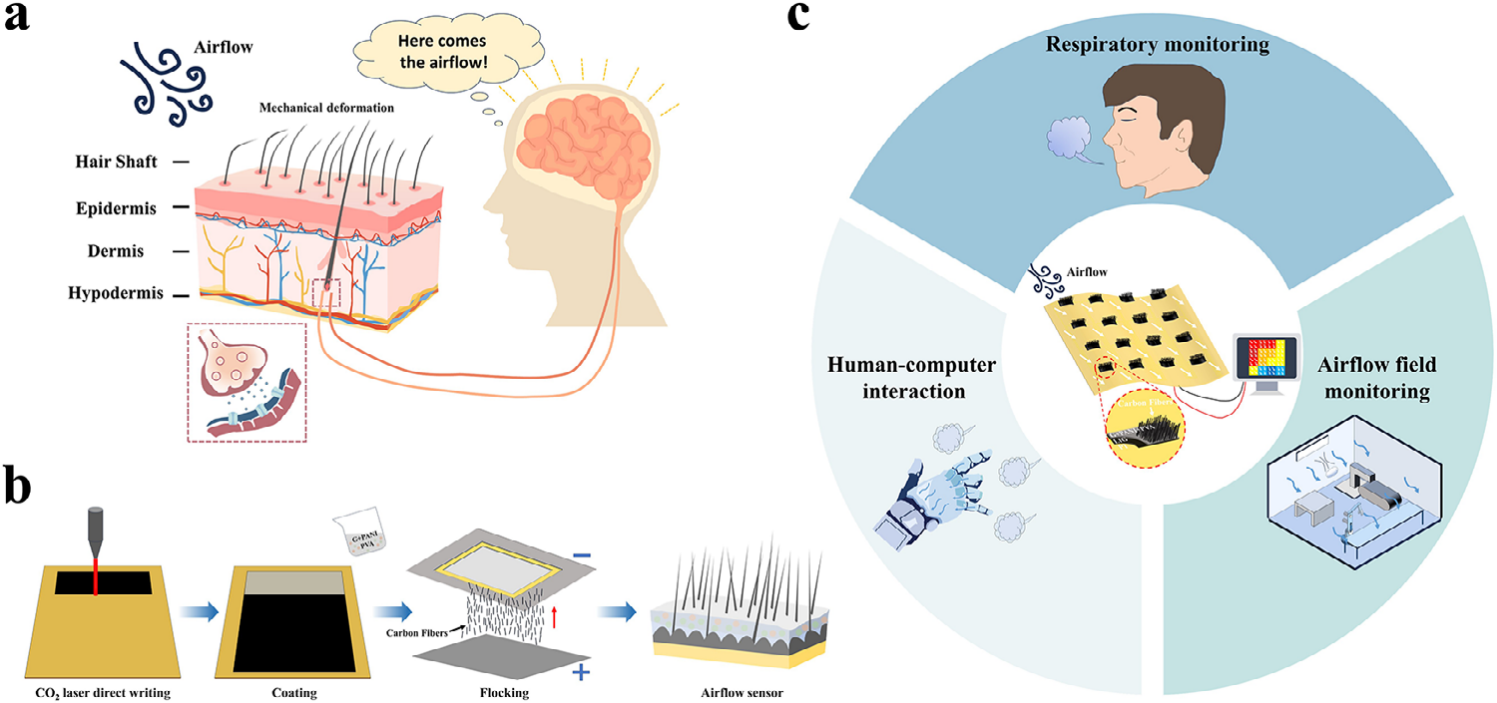
Human hair sensor system and hair‐like flexible airflow sensor array design. a) Schematic illustration of the structure and features of human skin. b) Schematic illustration of the airflow sensor fabrication process. c) Applications of the airflow sensor array in typical areas.

**Figure** **1**a illustrates the structure and characteristics of the human skin and the basic mechanism by which it senses airflow. Human skin is primarily composed of the epidermis, dermis, and subcutaneous tissue. Human hair can be divided into the hair shaft and the hair follicle. The hair follicle is situated within the dermis, encircled by numerous receptors, while the hair shaft extends outward through pores in the epidermis. The mechanism of human skin perceiving airflow can be simply elucidated as follows: when airflow interacts with the skin, the hairs deform under the pressure of the airflow. Receptors surrounding the hair follicles accurately detect these deformations and transmit the sensory information to the brain through the nervous system. With the distribution of hairs across the skin's surface, the human body can perceive and distinguish airflow intensity and direction accurately from various locations. Inspired by this, a hair‐like flexible airflow sensor modeled after the structure of human skin is designed and prepared. The fabrication process is shown in Figure 1b. First, laser‐induced graphene (LIG) is produced by directly writing on polyimide (PI) films with a carbon dioxide laser. Next, the LIG is coated with the prepared conductive adhesive. The carbon fibers are charged using a desktop electrostatic flocking device and propelled toward the grounded substrate due to the attraction of opposite charges. The carbon fibers are firmly fixed in the adhesive. After drying and curing, the flexible airflow sensor with a hair‐like surface structure is prepared. This composite‐structured sensor consists of a conductive layer, a bonding layer, and a fluff layer. The unique advantages of flexibility and easy installation make it deployable in complex environments and on irregular surfaces for large‐area airflow sensing. The efficient dynamic sensing capability of the airflow sensor makes it suitable for human respiration monitoring, environmental airflow analysis, human‐computer interaction, and other fields, demonstrating significant potential for widespread use.

**Figure 2:**
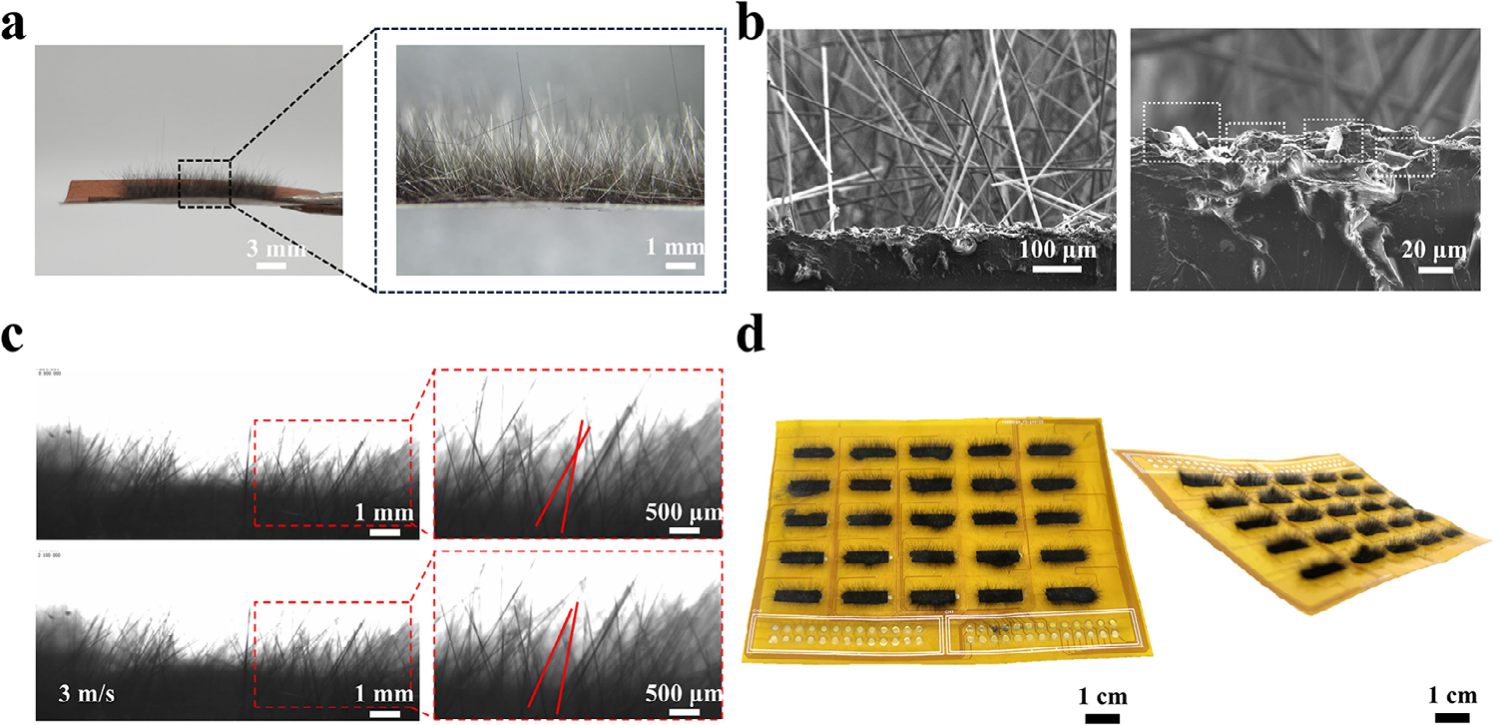
Mophorlogies and characterization of the airflow sensor. a) Side‐view photographs of the airflow sensor. The inset optical microscope image showing the fluff‐like surface topography. b) SEM images of the airflow sensor. c) Optical micrograph of vibrating carbon fibers in the fluff layer under airflow. d) Photographs of a hair‐like flexible airflow sensor array.

**Figure** **2**a shows the photographs of the airflow sensor, clearly illustrating its hair‐like surface morphology. The fluff morphology can be prepared on the substrate surface by the electrostatic flocking technique. A large number of carbon fibers are uniformly implanted by electrostatic flocking and are firmly fixed to the substrate. The optical microscope image reveals that the azimuth angles between carbon fibers and the substrate exhibit a certain degree of randomness. Most carbon fibers are fixed nearly vertically on the substrate, with only a few carbon fibers falling. Due to the different azimuth angles and small spacing between them, a large number of carbon fibers are in contact with each other. Figure 2b shows the cross‐sectional SEM image of the airflow sensor, which reveals its composite structure comprising a PI film base layer, an LIG conductive layer, a bonding layer of mixed polyvinyl alcohol (PVA), polyaniline (PANI), and graphene, and a fluff layer of carbon fibers. The striped morphology (Figure S1a, Supporting Information) and porous structure (Figure S1b, Supporting Information) of LIG not only provide more bonding sites for carbon fibers, but also provide enhanced conductive paths for the conductive layer. The Raman spectrum of LIG (Figure S2, Supporting Information) shows three distinct peaks, D peak near 1350 cm^−1^, G peak near 1580 cm^−1^ and 2D peak at 2700 cm^−1^, which are the typical peaks of graphene.^[^ ^29^, ^30^, ^31^ ^]^ Due to the thin conductive layer and the bonding layer in the sensor, no distinct boundary between these layers is visible in the SEM image. The electrostatically implanted carbon fibers penetrate to varying depths within the substrate. Some carbon fibers pass directly through the bonding layer to contact the conductive layer, while others remain embedded within the bonding layer, forming a composite conductive network that includes connections between carbon fibers, the conductive layer, the bonding layer, and among the layers themselves, establishing a foundation for the sensor's high performance. Figure 2c shows the airflow sensor under airflow. Similar to human hair, the carbon fibers in the fluff layer constantly vibrate under the influence of airflow. The dense fibers undergo continuous contact and separation, providing a unique response mechanism that supports the sensor's efficient airflow sensing capability. By conducting airflow field visualization observations in the air duct, this sensor causes less disturbance to the airflow field compared with commercial sensors (Figure S7, Supporting Information). Figure 2d shows the array of hair‐like flexible airflow sensors integration. The efficient, convenient, and controllable manufacturing process of the airflow sensor enables it to be easily integrated into an array, while the substrate's flexibility enables conformal sensing on irregular surfaces. The sensor array configuration can be tailored to meet the specific requirements of diverse airflow field monitoring applications, demonstrating adaptability across various environments.

**Figure 3:**
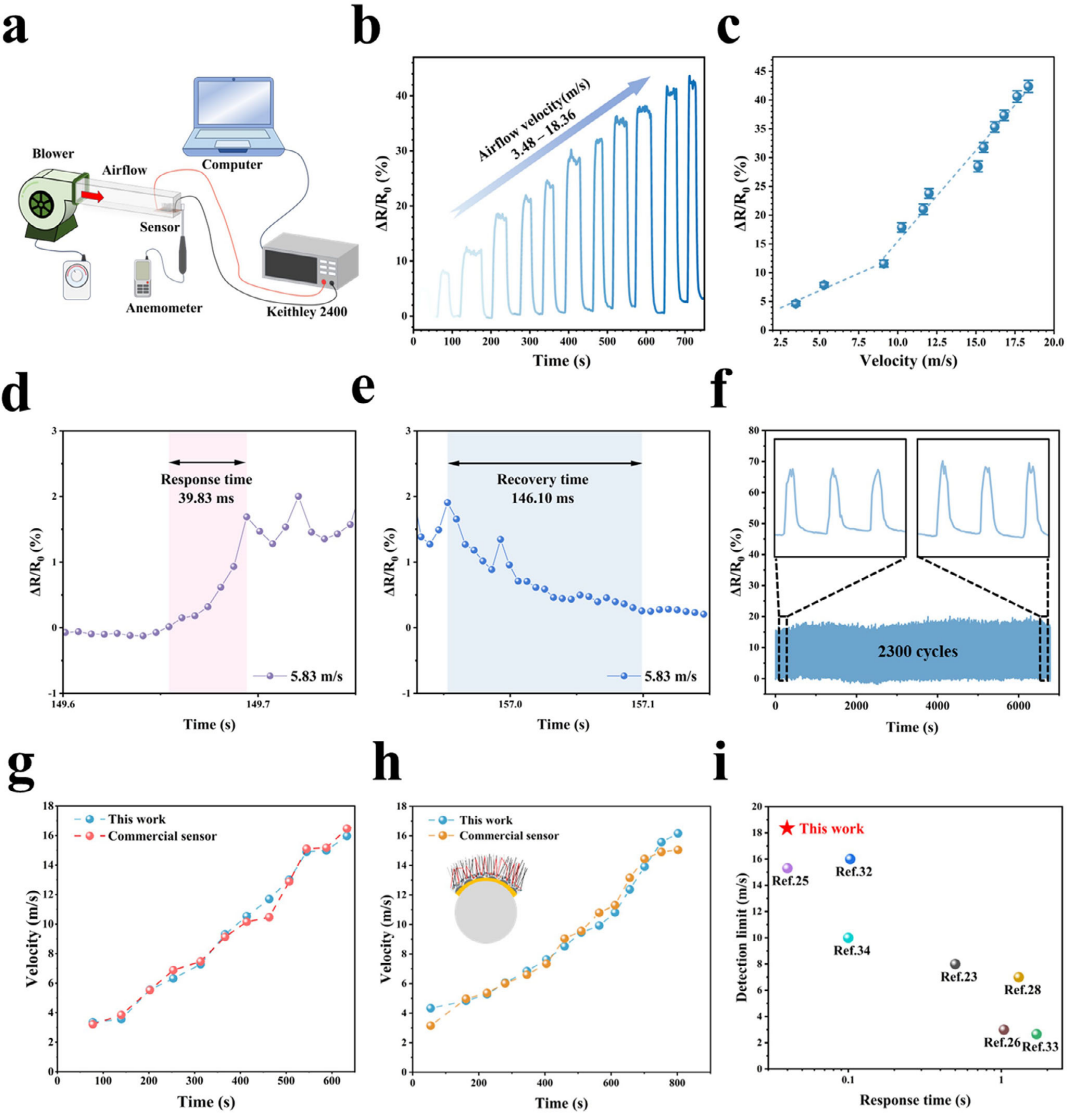
Performance of the airflow sensor. a) Schematic illustration of the platform for the airflow sensing test. b) The response curves of the airflow sensor against different airflow velocities. c) The corresponding relationship between ΔR/R_0_ and airflow velocity of the airflow sensor. d) and e) Response time and recovery time of the airflow sensor at an airflow velocity of 5.83 m s^−1^. f) ΔR/R_0_ response curves of the airflow sensor under 2300 airflow on/off cycles at an airflow velocity of 10 m s^−1^. g) Real‐time airflow velocity monitoring comparison between the fabricated sensor deployed on flat surface and commercial sensor. h) Real‐time airflow velocity monitoring comparison between the fabricated sensor deployed on a curved surface (0.02 mm^−1^) and commercial sensor. i) The response time and upper detection limit of the airflow sensor in comparison with hair‐structured airflow sensors reported in the literature.^[^ ^23^, ^25^, ^26^, ^28^, ^32^, ^33^, ^34^ ^]^

**Figure** **3**a shows the setup for the airflow sensing test. The prepared sensor exhibits excellent responsiveness across varying airflow velocities. The ΔR/R_0_ response curves for airflow velocities ranging from 3 to 18 m s^−1^ are shown in Figure 3b. Due to blower limitations, the lowest tested velocity is 3.48 m s^−1^. When airflow at a certain velocity blows towards the sensor, the ΔR/R_0_ value rapidly increases to a peak and then stabilizes within a range. As airflow velocity increases, the ΔR/R_0_ value also rises, showcasing the sensor's broad detection range and its capability to effectively distinguish between different airflow velocities. Sensitivity is a critical parameter for sensors. Here, sensitivity (S) is calculated by the following formula^[^ ^32^ ^]^:

(1) $$S=\frac{\delta \left(\frac{\Delta R}{R_{0}}\right)}{\delta v}$$

Here, S represents the sensitivity, ∆R represents the resistance change, R_0_ represents the initial resistance of the sensor, and v represents the airflow velocity.

The slope of the ΔR/R_0_ variation curve (Figure 3c) corresponding to different airflow velocities is the sensitivity of the sensor (S). The curve is divided into two distinct sections: for airflow velocity below 9 m s^−1^, the sensitivity of the sensor is 1.19% s m^−1^, while for airflow velocity above 9 m s^−1^, the sensitivity of the sensor increases to 3.16% s m^−1^. The carbon fibers’ strength and firm adhesion ensure rapid response and recovery under airflow. As shown in Figure 3d,e, the airflow sensor exhibits a response time of 39.83 ms and a recovery time of 146.10 ms at an airflow velocity of 5.83 m s^−1^. Response time is defined as the time required for ΔR/R_0_ to reach 90% of the response value after airflow initiation, while recovery time is the time required for it to reduce by 90% after airflow cessation. Figure 3f shows the cyclic variation curves of the airflow sensor after 2300 consecutive airflow on/off cycles. The resistance signal remains stable throughout the test, with no significant degradation observed in the curves during the cycle. This highlights the airflow sensor's excellent stability and reliability, supporting long‐term, repeated usage. Figures 3g,h demonstrate the ability of the sensors in this work to accurately and reliably detect airflow velocity on flat and curved surfaces, respectively. In real‐time wind speed detection tests, the average relative error between the detection values of the sensors in this work and those of commercial sensors is less than 3.8% (on a flat surface) and 6.6% (on a curved surface). Compared with previously reported hair‐structure airflow sensors, the airflow sensor in this paper achieves faster response time, a wider detection range (Figure 3i), and a more efficient fabrication method (Table S1, Supporting Information).^[^ ^23^, ^25^, ^26^, ^28^, ^32^, ^33^, ^34^ ^]^

**Figure 4:**
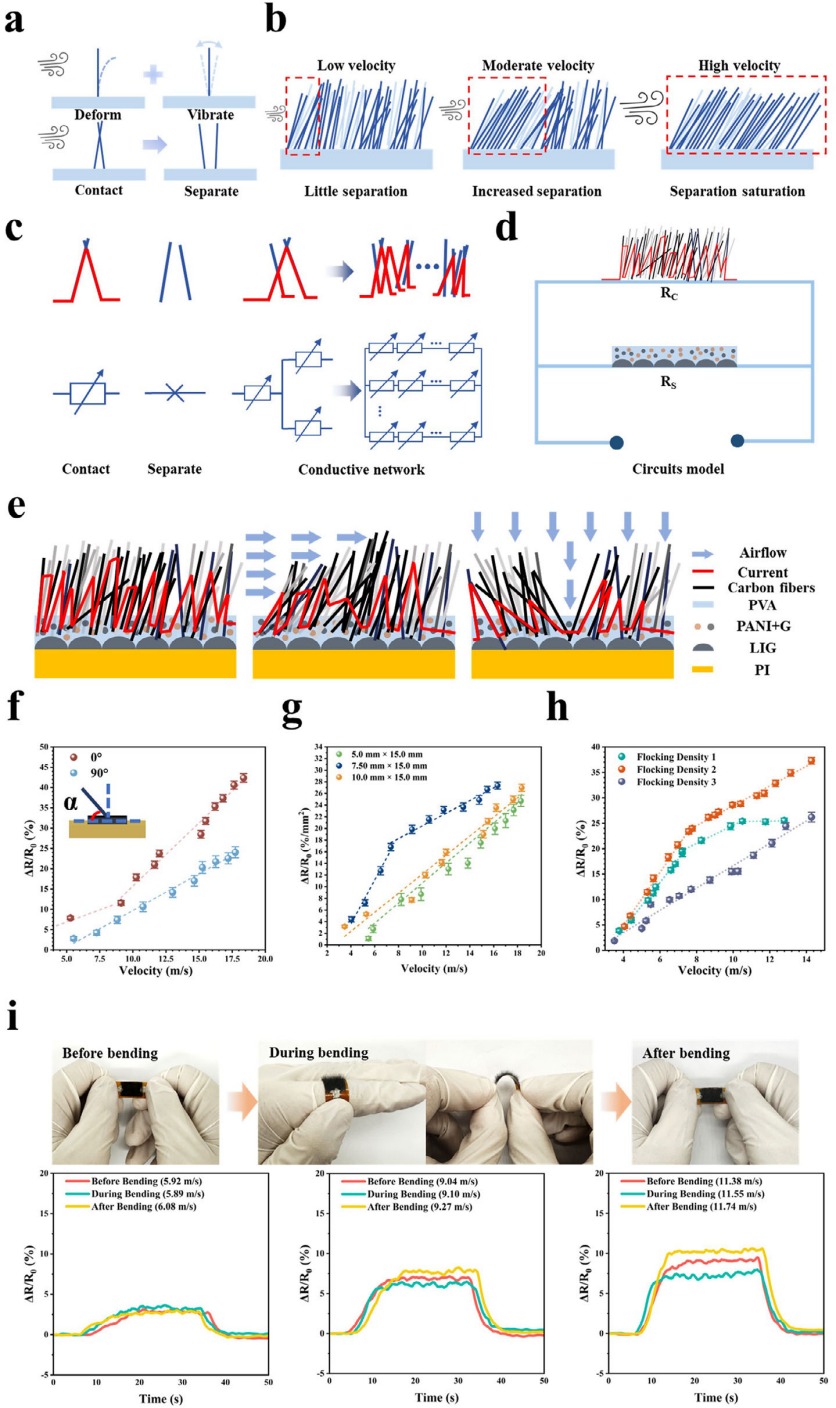
Working mechanism of the airflow sensor. a) Illustration of the carbon fiber response model under the airflow. b) Illustration of the carbon fibers separation of the fluff layer under different velocities of airflow. c) Schematic diagram of the carbon fiber conductive network model. d) Schematic diagram of the circuit model of the sensor. e) Schematic diagram of the current flow of the airflow sensor under no airflow (left), 0° airflow (middle), and 90° airflow (right). f) ΔR/R_0_ of the sensor under airflow of 0° and 90° respectively. g) ΔR/R_0_ of the sensor under different sizes. h)ΔR/R_0_ of the sensor at different flocking densities. i) ΔR/R_0_ of the sensor before, during, and after bending.

To elucidate the sensing mechanism of the sensor more comprehensively, the carbon fiber mechanical response model under airflow, shown in **Figure** **4**a is established. The carbon fiber in the fluff layer can be simplified as a cantilever beam, so the carbon fiber will deform and vibrate under airflow (Figure S5, Supporting Information). The first‐order resonant frequency of the carbon fibers in the fluff layer is around 3 kHz. The dense carbon fibers cause the airflow effect to be dispersed to a certain extent, resulting in the vibration of the carbon fibers usually manifested as bending vibration (Figure S6, Supporting Information). The contact force between carbon fibers is influenced by factors such as stiffness, azimuth angle, and gravity, resulting in differences in contact force between different carbon fibers. When the pressure of airflow exceeds their contact force, the carbon fibers separate. Figure 4b shows the carbon fibers separation of the fluff layer under different velocities of airflow. When the airflow velocity is low, only a small amount of carbon fibers are affected, resulting in little separation. As the airflow velocity increases, the number of affected carbon fibers increases, leading to an increase in carbon fibers separation. Once the airflow velocity reaches a certain value, the majority of carbon fibers in the fluff layer are affected, reaching saturation. It means that even if the airflow velocity continues to increase, it has little effect on the carbon fibers separation (Video S2, Supporting Information). To analyze the effect of carbon fiber contact and separation on the sensing signal, a conductive network model as shown in Figure 4c was established. When conductive carbon fibers are in contact, they can be equivalent to the a resistor composed of their own resistance and contact resistance. When carbon fiber separates, it is equivalent to a circuit break. In the fluff layer, numerous carbon fibers are in contact with each other, which can be equivalent to a certain number of resistors connected in parallel.

Figure 4d shows the circuit model of the sensor. When current flows through the sensor, it is primarily conducted through two paths: one through the conductive layer and the bonding layer of the sensor, and the other through the fluff layer composed of carbon fibers. Therefore, the circuit model of the airflow sensor can be simplified to a parallel connection between the resistance (R_S_) of the electrode and bonding layers, and the resistance (R_C_) of the fluff layer. The total resistance (R_t_) of the sensor is calculated by the following formula:

(2) $$R_{t}=\frac{R_{S}\cdot R_{C}}{R_{S}+R_{C}}=\frac{R_{C}}{1+R_{C}/R_{S}}$$

The resistance (R_C_) of the fluff layer is mainly affected by the conductive paths formed by carbon fibers in contact. The resistance (R_CP_) of the contact path mainly consists of the resistance of the carbon fiber itself and the contact resistance:

(3) $$R_{CP}=\rho_{c}\cdot \left(\frac{A_{c}}{\rho_{r}A_{r}}+\frac{L}{A}\right)$$

Here, ρ_c_ represents the volume resistivity, ρ_r_ represents the contact resistivity, L represents the effective length, A_c_ represents the contact area, A_r_ represents the actual contact area, and A represents the cross‐sectional area of carbon fiber. Assuming that there are n conductive paths formed by carbon fibers in contact in the fluff layer, the equivalent resistance (R_C_) of the fluff layer is:

(4) $$R_{C}={\left(\frac{1}{R_{CP1}}+\frac{1}{R_{CP2}}+\cdots \frac{1}{R_{CPn}}\right)}^{-1}$$

Given the high density of carbon fibers in the fluff layer, most fibers are in contact with each other. R_S_ is much higher than R_C_, making R_t_ approximately equal to R_C_, where current mainly flows through the carbon fibers. Sustained airflow increases the separation among carbon fibers, rapidly reducing the conductive paths in the fluff layer. Therefore, R_C_ increases, which in turn increases R_t_. The degree of resistance change correlates with airflow velocity, as a higher velocity induces greater separation, leading to a larger resistance increment (ΔR_C_) of the fluff layer increases and a higher resistance change rate (ΔR/R_0_). This mechanism underpins the sensor's ability to detect airflow. Figure 4e shows the schematic diagram of the sensor's airflow sensing mechanism. When the airflow is off, most carbon fibers are in contact, forming numerous conductive paths. When the airflow is blowing, the carbon fibers are forced to vibrate, causing rapid separation and a reduction in the initial conductive paths. The current is then forced through the bonding layer and conductive layer, which exhibit relatively higher resistance. Figure 4f shows the ΔR/R_0_ variation curves of the sensor at 0° and 90° airflow. When the airflow is blowing and the angle between the airflow and the sensor is 90°, the sensitivity is 1.81% s m^−1^, while when the angle is 0°, the sensitivity is 1.19% s m^−1^ (< 9 m s^−1^) and 3.16% s m^−1^ (> 9 m s^−1^). The fluff layer, composed of a large number of carbon fibers arranged at specific angles to one another, forms a unique porous structure with numerous carbon fiber contact points. The separation of carbon fibers caused by airflow reduced the number of contact points, leading to an increase in resistance. This resembles a percolation effect in conductive composites, where changes in the number of conductive particles result in alterations to the material's conductivity. Due to differences in contact force and the density of carbon fibers, the required airflow velocity for separation varies, resulting in a segmented sensitivity of the sensor. When the airflow velocity exceeds a certain value, there is a significant change in sensitivity, which is more obvious when the airflow blows at 0°. At 0° airflow, the carbon fibers in the front obstruct those behind, reducing the effective airflow and, consequently, lowering sensitivity compared to 90°. As airflow velocity increases, the pressure on the surface of the carbon fibers intensifies. At 0°, the carbon fibers exhibit larger deformation and vibration, enhancing sensitivity. Figure 4g shows the ΔR/R_0_ variation curves for different sensor sizes. When the angle of the airflow is 0° and the length of the airflow sensors is 15 mm, the sensor sensitivity per mm^2^ is 1.73% mm^−2^ s m^−1^ at a width of 5 mm, 3.77% mm^−2^ s m^−1^ (< 9.14 m s^−1^) and 1.63% mm^−2^ s m^−1^ (> 9.14 m s^−1^) at a width of 7.5 mm, and 1.64% mm^−2^ s m^−1^ at a width of 10 mm. Wider sensors have larger fluff layers that interact differently with airflow, affecting sensitivity. In terms of the sensor's width, when it is too small, the amount of carbon fibers separated by airflow in the fluff layer is reduced, resulting in lower sensitivity. On the contrary, when the width is too large, due to the obstruction of carbon fibers to the airflow, the sensor sensitivity is low. Until the airflow velocity increases, the influence of the obstruction is weakened, and the sensor sensitivity is significantly improved. The density of carbon fibers implanted into the bonding layer by electrostatic flocking is described as the flocking density. The flocking density will not only affect the surface topography of the sensor, but also affect the sensitivity of the sensor. Figure 4h shows the variation curve of ΔR/R_0_ for flocking density from low to high. With the increase of the flocking density, the carbon fibers in the fluff layer gradually change from sparse to dense (Figure S8, Supporting Information). The higher the flocking density, the more carbon fibers per unit area, and thus the more carbon fibers in contact with each other. The airflow sensor with low flocking density (flocking density 1) shows a gradual flattening of the ΔR/R_0_ variation curve of the airflow sensor, with a gradual decrease in sensitivity up to 4.56% s m^−1^ (< 7 m s^−1^). The airflow sensor with moderate flocking density (flocking density 2) has the highest sensitivity of 5.25% s m^−1^ (< 8 m s^−1^). The airflow sensor with high flocking density (flocking density 3) has a sensitivity of 2.19% s m^−1^. When the flocking density is low, the airflow sensor has a good response under relatively low‐speed airflow, but due to the small amount of carbon fibers, as the airflow speed rises, the sensitivity of the sensor gradually decreases, and eventually becomes saturated. When the flocking density is high, the number of carbon fibers per unit area is large. The deformation of the carbon fibers is limited by other carbon fibers when the airflow blows, so fewer carbon fibers are separated from each other, resulting in a decrease in the sensitivity of the sensor. Figure 4i shows the photograph and the airflow sensing performance of the airflow sensor before and after bending. The excellent flexibility of the sensor allows it easy to bend to large angles and recover. Furthermore, the sensor maintains stable airflow sensing across velocities during and after bending (Figure S10, Supporting Information). When sensors are arranged on surfaces with different curvatures, in the absence of air flow, as the surface curvature increases, the carbon fibers originally in contact in the sensor's fluff layer separate, resulting in an increase in sensor resistance (Figure S11, Supporting Information). This further confirms the influence of the contact and separation of carbon fibers on the sensor's resistance value. This allows the sensor to be easily arranged on various surfaces while still detecting airflow effectively. Additionally, the ΔR/R_0_ of the sensor decreases progressively at the same airflow velocity from 25 °C to 120 °C (Figure S12, Supporting Information), indicating the fluff layer's resilience to temperature effects due to its role as the primary sensitive element.

**Figure 5:**
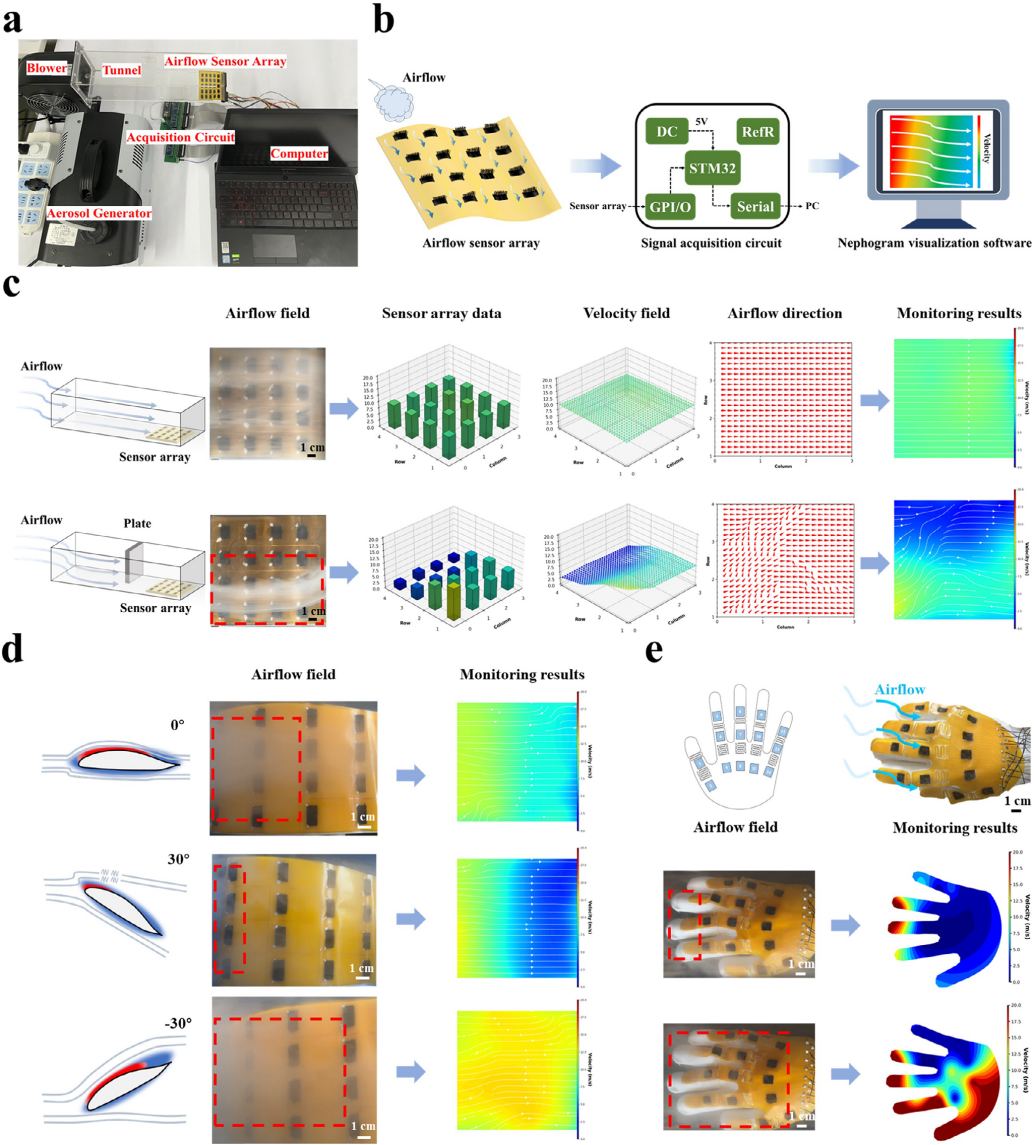
Applications of the airflow sensor array. a) Schematic illustration of the airflow field monitoring test platform. b) Schematic illustration of an airflow field real‐time monitoring system. c) Images of the airflow field, the vector field reconstruction and monitoring results at an airflow velocity is 10 m s^−1^. d) Images of the airflow field and monitoring results on the surface of the airfoil of NACA 5‐H‐20 at 0°, 30° and ‐30°, respectively, when the airflow velocity is 10 m s^−1^. e) Photographs of gloves integrated with sensor arrays and the airflow field monitoring results of the gloves’ surface.

To validate the large‐area airflow sensing capability of the hair‐like flexible airflow sensor, a test platform is built as shown in **Figure** **5**a. The sensor is integrated into a 4 × 4 array with a size of 100 mm × 100 mm. Each sensor measures 15 mm × 7.5 mm and is distributed at equal intervals, with a spatial resolution of 30 mm × 22.5 mm. A large‐area airflow real‐time sensing system is shown in Figure 5b. The response data of the sensor array is collected by a signal acquisition circuit, and the monitoring results are displayed in real‐time by nephogram visualization software. The response data of the sensor array reflects the magnitude of the airflow velocity at different positions. Airflow direction is inferred by assuming that streamlines follow the gradient of decreasing velocity, subject to prescribed boundary conditions. To further confirm the reliability of the monitoring results, a smoke generator is used to make the actual airflow field visible. As shown in Figure 5c, the sensor array is placed inside the duct, under two test conditions: 1) no block plate; 2) a block plate covering half the duct width. In test condition 1, the airflow remains stable, with uniform velocity and direction, as reflected in the monitoring results. In test condition 2, the block plate causes airflow to converge in the unblocked area, sharply increasing velocity there while reducing it in the blocked region, which is accurately monitored by the sensor array. To further verify the resolution of the sensor array for changes in the airflow field, blocks were placed at different positions in front of the sensor array, and the sensor array could effectively identify the changes in the airflow field (Figure S13, Supporting Information). To demonstrate the sensor array's ability to identify different airflow fields, unsteady airflow field tests are conducted on the sensor array. By adjusting the velocity and placing baffles during the test to change the airflow field state, the sensor array can effectively identify this change (Video S4, Supporting Information). Figure 5d shows the excellent conformal monitoring capability of the hair‐like flexible airflow sensor array. The airflow sensor array (100 mm × 200 mm) is placed on the surface of NACA 5‐H‐20 airfoil, and monitors the surface airflow field at three different angles of 0°, 30°, and –30°. At 0°, airflow is strongest at the leading edge, weakening towards the rear. The actual monitoring results show that the front section is a high‐velocity area, while the rear section has a low velocity. When the airfoil is at 30°, the airflow at the front is stronger, and the airflow separation occurs, with weaker airflow at the rear. The monitoring result shows the high velocity rate area at the front, and the streamlines gather at the rear. When the airfoil is at –30°, the entire surface of the airfoil is affected by the airflow. The monitoring results show a large range of high velocity areas and chaotic streamlines. The reliability of the hair‐like flexible airflow sensor array in real‐time dynamic monitoring of the airflow field and its ability to monitor the airflow field on various surfaces are demonstrated. The fast, simple, and customizable fabrication of the hair‐like flexible airflow sensor array enables not only conformal monitoring, but also adaptation to specific applications. Here, a hand‐shaped airflow sensor array with 14 sensors is fabricated and integrated onto a glove. In order to minimize signal drift caused by bending strain during hand movements, the sensor units are positioned between the finger joints, and the structure at the finger joints is designed to conform to bending. By applying airflow to different parts of the glove, the airflow sensor array allows rapid, accurate detection of airflow on individual fingers and across the glove's surface (Figure 5e). This provides intelligent devices such as wearable devices and intelligent robots with efficient airflow perception manner while retaining a similar appearance to human hair. The above application test, as a preliminary verification, demonstrated that the sensor can be easily integrated into an array and effectively detect changes in airflow within the area. Through this efficient preparation process and excellent performance, the sensor array can be easily expanded to a large‐area array and achieve effective airflow perception, demonstrating great application potential in large‐area airflow perception.

## 3 Conclusion

In summary, drawing inspiration from human skin, hair‐like flexible airflow sensors are developed using laser direct writing and electrostatic flocking technologies. PI film serves as the substrate, on which the loose and porous LIG conductive layer is prepared by laser direct writing. Numerous carbon fibers are firmly fixed on the substrate by electrostatic flocking, forming a hair‐like structure similar to human hair with high sensitivity to airflow. The sensors efficiently and reliably sense airflow in real time over large areas by leveraging resistance changes driven by variations in the contact area of carbon fibers under airflow. Due to the hair‐like structure, the fabricated sensors exhibit high sensitivity (5.25% s m^−1^), fast response time (39.83 ms), a wide detection range (3.48 – 18.36 m s^−1^), and excellent cyclic stability (2300 cycles). Additionally, the remarkable flexibility and the customizable fabrication process enhance its versatility for diverse applications. As proof of application, several potential applications of the sensors are demonstrated, including real‐time airflow sensing in the air duct, real‐time airflow sensing on the airfoil surface, and interactive airflow sensing via a smart glove. The sensors excellent performance, flexibility, and ease of customization make it highly suitable for a wide range of applications, including environmental monitoring, wearable devices, intelligent robots, aerodynamic design, and tactile sensing.

## 4 Experimental Section

### Materials

Polyvinyl alcohol (PVA, molecular mass 205 000, Shanghai Macklin Biochemical Technology Co., Ltd., China), multilayer graphene (G, analytically pure, Kaisa New Materials Co., Ltd., China), conductive polyaniline (PANI, analytically pure, Kuer Chemical Technology Co., Ltd., China), polyimide film (PI, thickness = 25 µm, Kapton, Ronghui Co., Ltd., China), carbon fibers (length = 3 mm, Toray Co., Ltd., Japan), anhydrous ethanol (analytically pure, Sinopharm Chemical Reagents Co., Ltd., China), deionized water.

### Fabrication of the Airflow Sensor Array—Preparation of the LIG

After the polyimide film was cleaned with anhydrous ethanol and dried, the surface of the polyimide film was irradiated with a CMH0604‐B‐CO_2_ laser (Guangdong Dazu Yueming Laser Group Co., Ltd., China) to produce laser‐induced graphene (LIG) (Figure S1, Supporting Information). The laser parameters are as follows: laser wavelength of 10.64 µm, laser spot diameter of 70 µm, laser power of 6.4 W, scanning speed of 100 mm s^−1^, scanning distance of 127 µm.

### Fabrication of the Airflow Sensor Array—Preparation of the Adhesive

6.5 g PVA was added to 50 ml deionized water and stirred at 500 RPM for 3 h in a water bath at 80 °C. Cool to room temperature, 0.4 g of PANI and 0.2 g of G were added, and stir at room temperature for 1 h at 300 rpm (Figure S2, Supporting Information).

### Fabrication of the Airflow Sensor Array—Electrostatic Flocking Process

A desktop electrostatic flocking device (Shenzhen Baoyi Model Design Co., Ltd., China) was used to conduct the electrostatic flocking process. 3 mm carbon fibers were ultrasonic dispersed with anhydrous ethanol and placed on a metal table and connected to the positive electrode. The regulated voltage was 16 500 V, and under the influence of the electrostatic field, the carbon fiber will accelerate almost vertically, and be uniformly implanted in the adhesive material. Solidifying at 80 °C for 2 – 3 h to obtain a high‐performance airflow sensor array with a hair‐like surface structure.

### Characterization

The surface morphology was investigated via field emission scanning electron mircroscopy (FE‐SEM, Merlin, ZEISS), the raman spectrum of the LIG was performed by Raman spectroscopy (LabRAM Aramis, HORIBA).

### Sensing Performance of the Sensor—Sensing Performance to Airflow of a Single Sensor

The test environment is: room temperature (25 °C – 27 °C), relative humidity (40% – 60%). The test scheme was to use a customized acrylic duct to access the outlet of a 120 W variable frequency centrifugal blower (Dongguan Shuangyi Electromechanical Co., Ltd., China). The airflow was generated by the blower, directed through a rectangular duct, and adjusted to achieve uniform and smooth flow. T The sample was placed at the rear of the duct, and the velocity of airflow was controlled by adjusting the blower speed. The airflow velocity was directly read by the AR866A digital thermal anemometer (Dongguan Wanchuang Electronic Products Co., Ltd., China). The sensor resistance change was measured with a digital source meter (Keithley 2400, Keithley Instruments, Inc., USA). The commercial sensor used for comparative testing was a hot‐wire airflow sensor (UAS 1000LP, Degree Controls, Inc., USA).

### Sensing Performance of the Sensor—Sensing Performance to Airflow Field of Sensor Array

The airflow sensor array was arranged in the duct and the surface of the object. The data was collected by the array acquisition circuit, and the airflow field data was visualized by the airflow field nephogram visualization software.

## Conflict of Interest

The authors declare no conflict of interest.

## Author Contributions

Y. Xie and F. Liu conceived and contributed the idea of the study, carried out experiments and wrote the manuscript. X. Wu contributed to experimental analysis and material characterization. Z. Wu and N. Lin contributed to the nephogram visualization software. Y. Luo and L. Lu provided experimental platform, completed the support materials and improved the manuscript.

## Supporting information



Supporting Information

Supplemental Video 1

Supplemental Video 2

Supplemental Video 3

Supplemental Video 4

Supplemental Video 5

Supplemental Video 6

Supplemental Video 7

## Data Availability

The data that support the findings of this study are available from the corresponding author upon reasonable request.
