# Supplementary material for: Hair‐Like Flexible Airflow Sensor for Large‐Area Airflow Sensing
Source: Adv Sci (Weinh). 2025 Sep 9;12(44):e10741. doi: 10.1002/advs.202510741 (PMC12667470; doi:10.1002/advs.202510741)
Supplement: Supplementary file 1 — Supporting Information [file ADVS-12-e10741-s008.docx]

**Supporting Information**

**Hair-like Flexible Airflow Sensor for Large-area Airflow Sensing**

*Yingxi Xie, Feilong Liu, Yongchao Luo*, Na Lin,Xiaohua Wu, Zeji Wu, Longsheng Lu*

Prof. Y. Xie, F. Liu, X. Wu, Z. Wu, Prof. L. Lu

School of Mechanical & Automotive Engineering, South China University of Technology, Guangzhou 510641, China

A.P. Y. Luo

School of Electronic And Information Engineering, Guangzhou City University of Technology, Guangzhou 510800, China

**E-mail**: luoyc@gcu.edu.cn

N. Lin

National Science and Technology Venture Capital Development Center, Beijing 100036, China

*Corresponding Author.


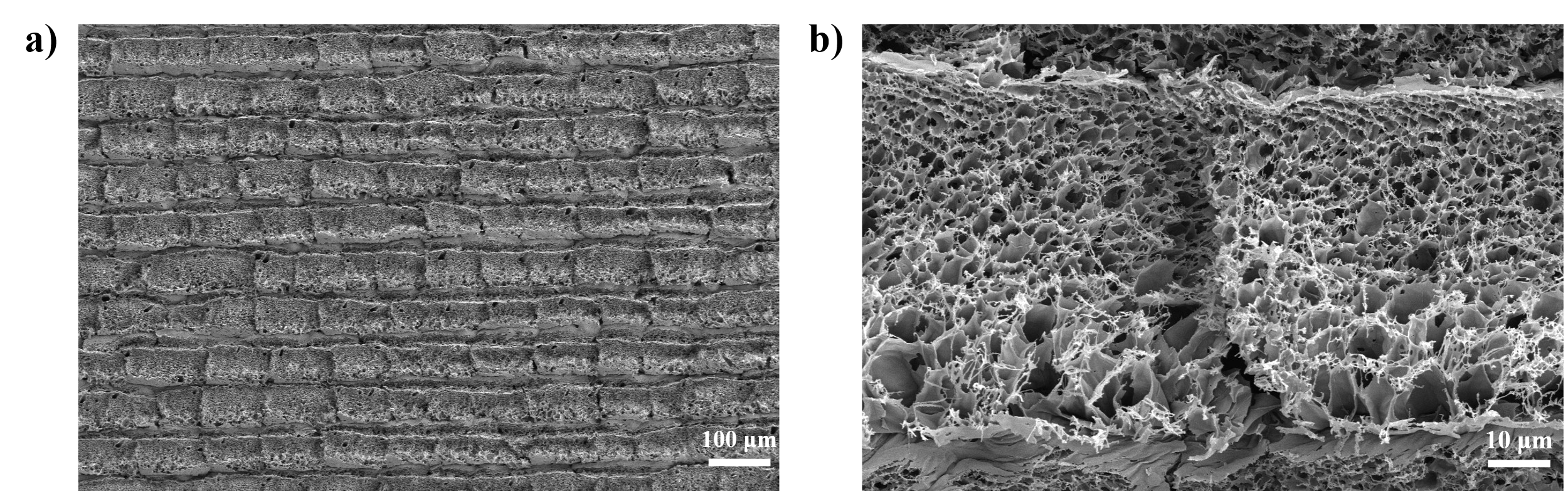


**Figure S1.** SEM imagess of the LIG. a) Striped morphology of the LIG. b) Loose and porous morphology of the LIG.





**Figure S2.** Raman spectrogram of LIG


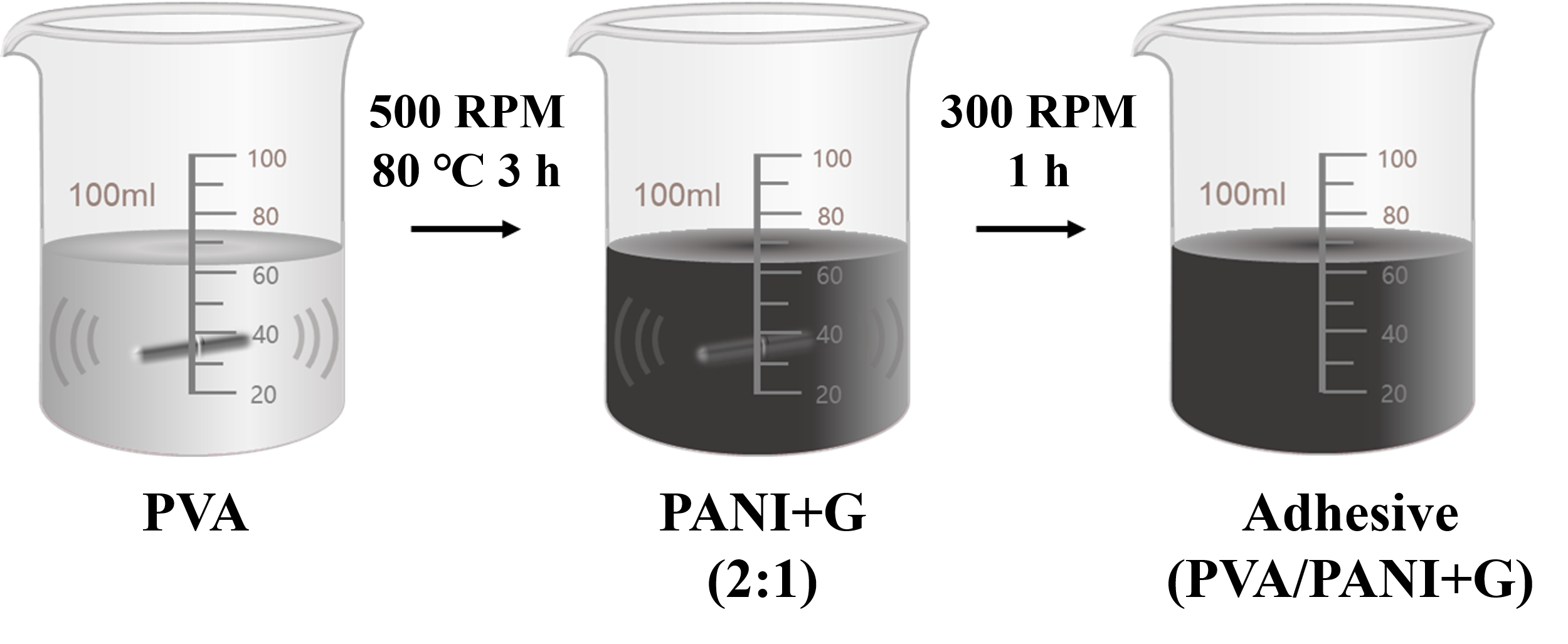


**Figure S3.** Illustrations of the preparation process of the adhesive (PVA/PANI+G).


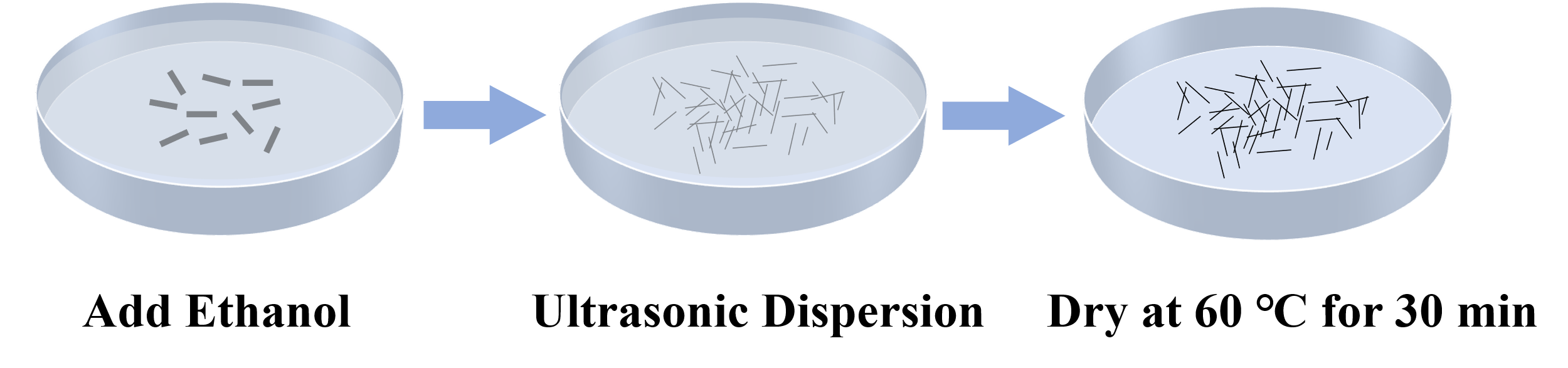


**Figure S4.** Illustrations of the treatment of carbon fibers


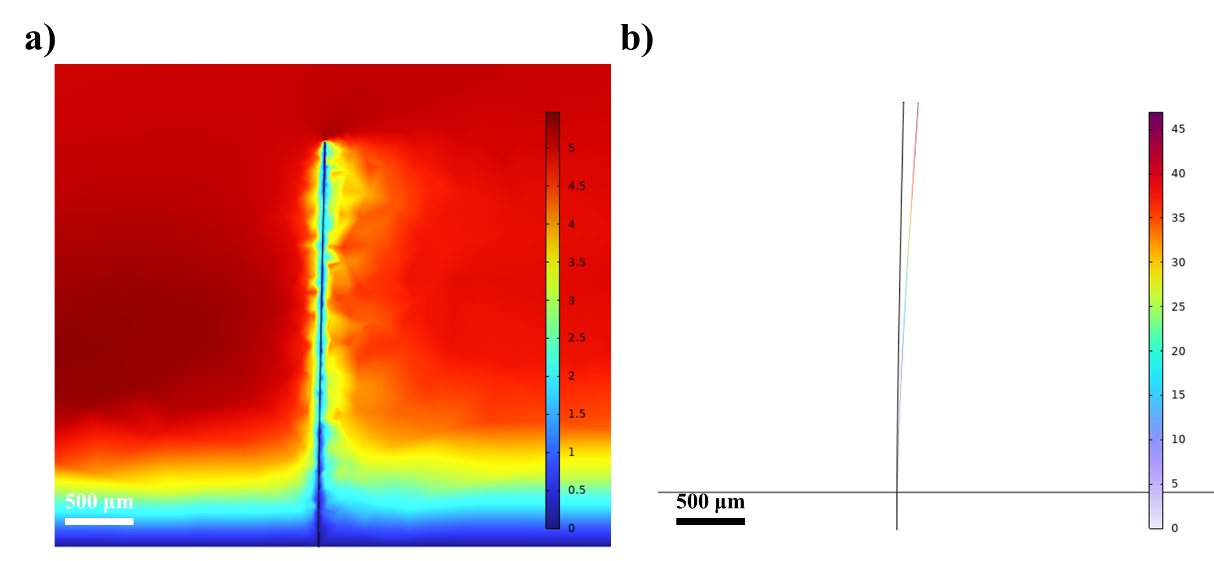


**Figure S5.** Static analysis of carbon fiber. a) Velocity cloud map at the cross section; b) Displacement cloud map at the cross section


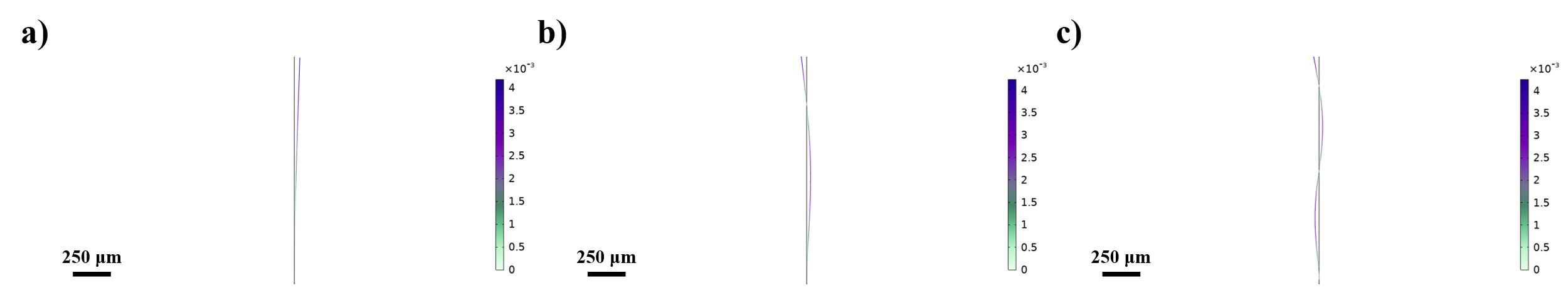


**Figure S6**. Modal analysis of carbon fiber. The mode shape at the characteristic frequency of a) 3520.5 Hz, b) 22062 Hz and c) 61772 Hz.


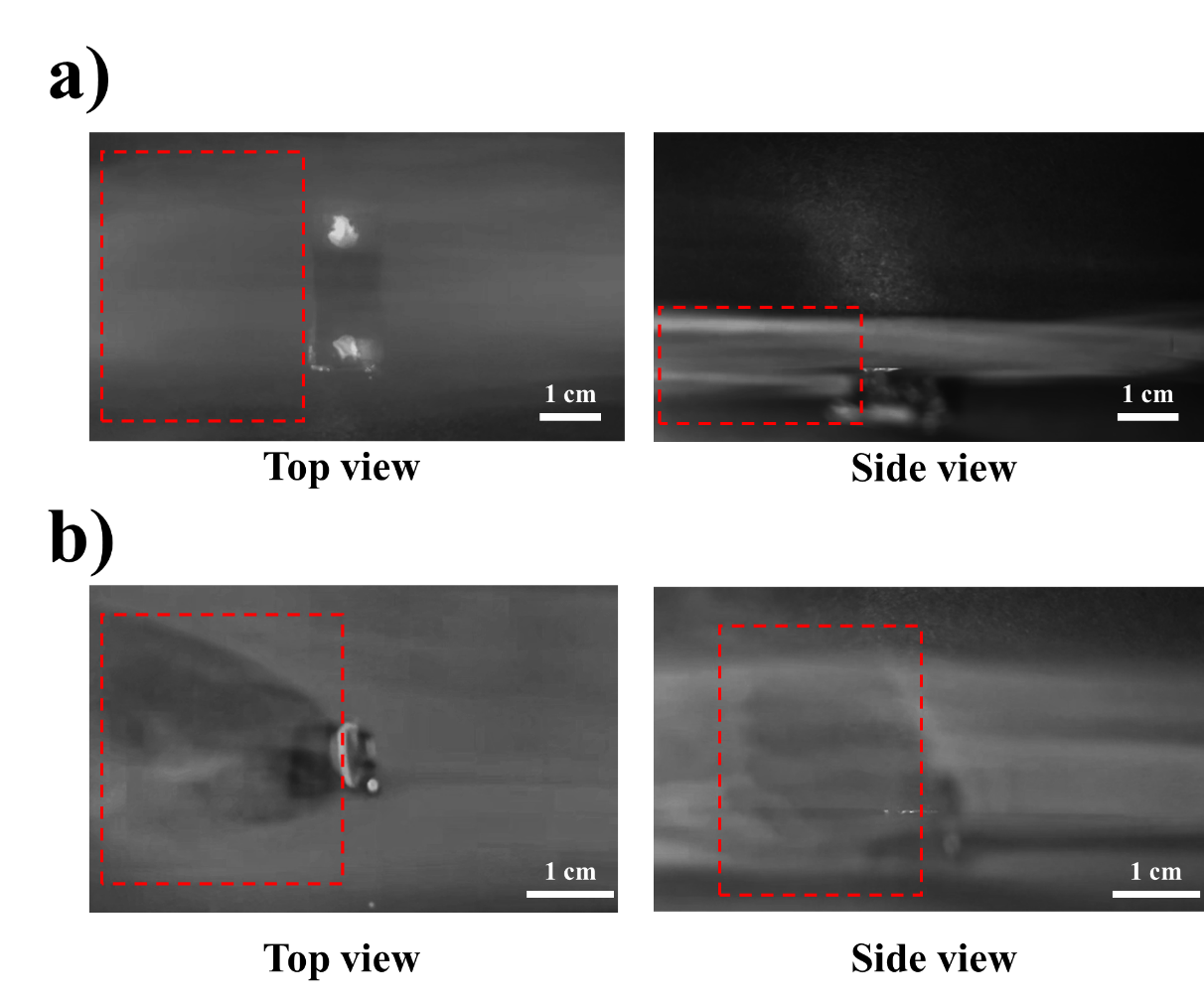


**Figure S7.** Comparison between the a) sensor in this work and b) commercial sensor for visualization of airflow field disturbances.


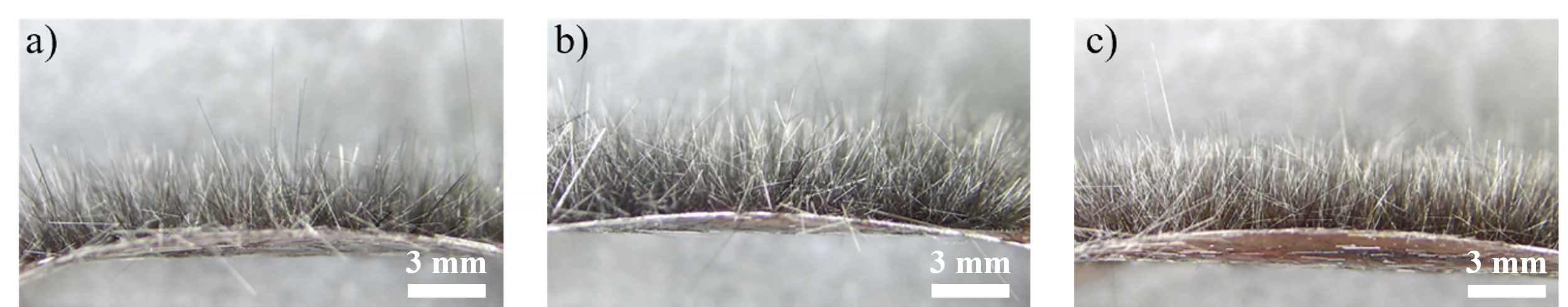


**Figure S8.** Photographs of different flocking densities sensors.


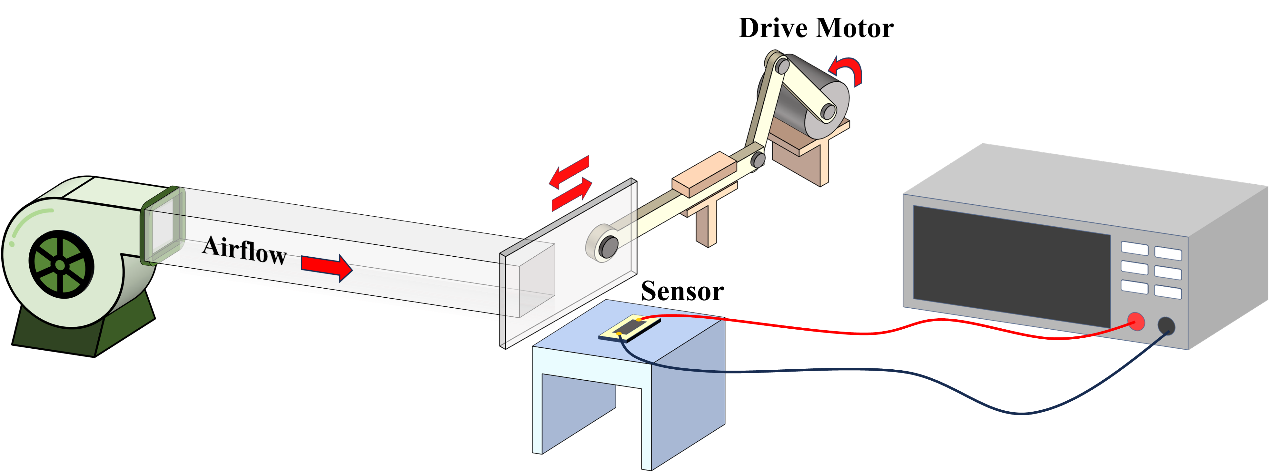


**Figure S9.** Schematic diagram of the airflow cycle testing platform.


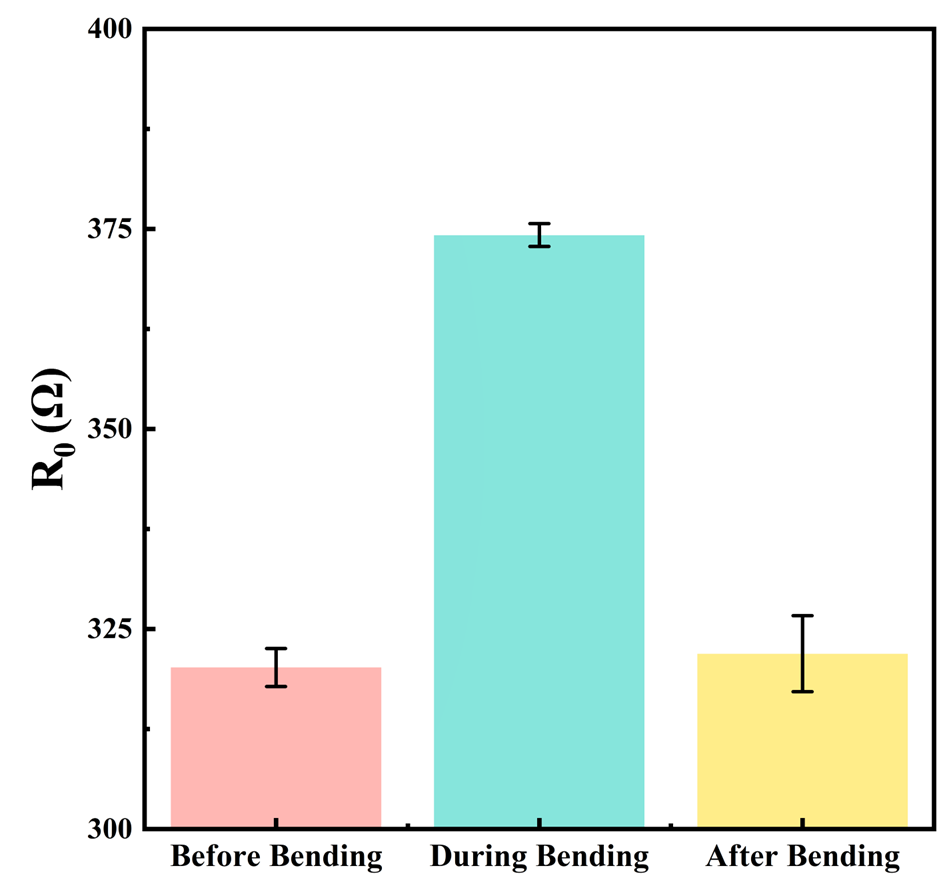


**Figure S10.** Initial resistance ($R_{0}$) of the sensor before, during and after bending.


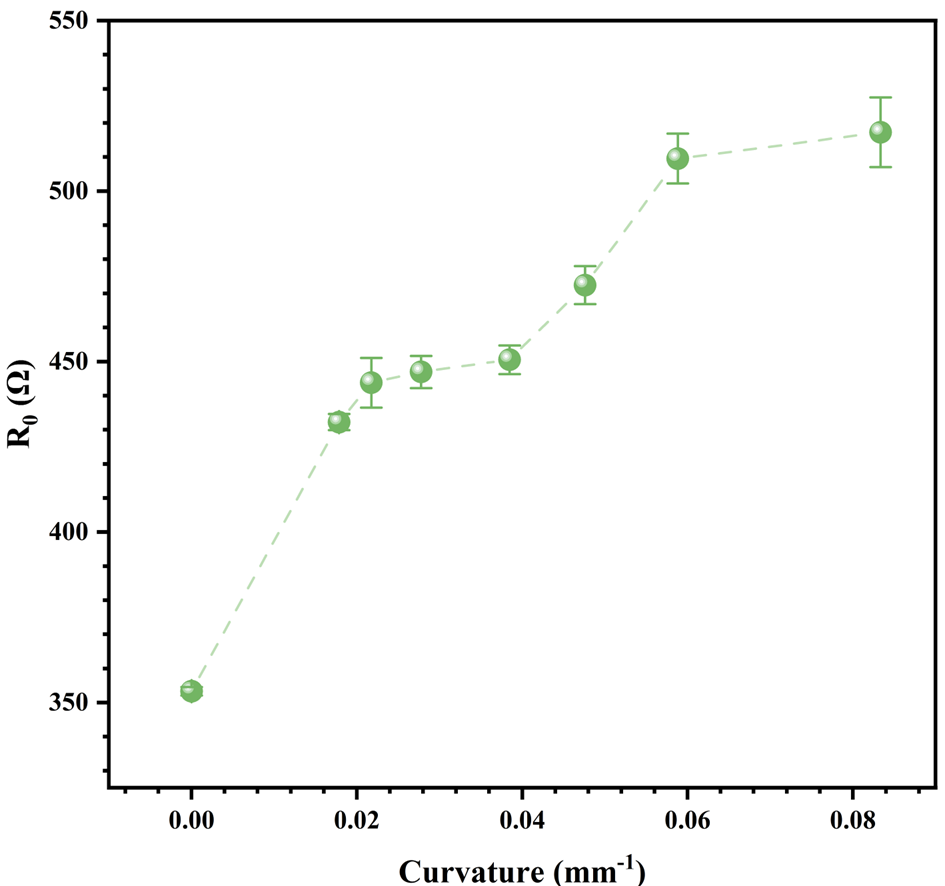


**Figure S11.** Resistance of the sensor under different curvatures (without airflow).


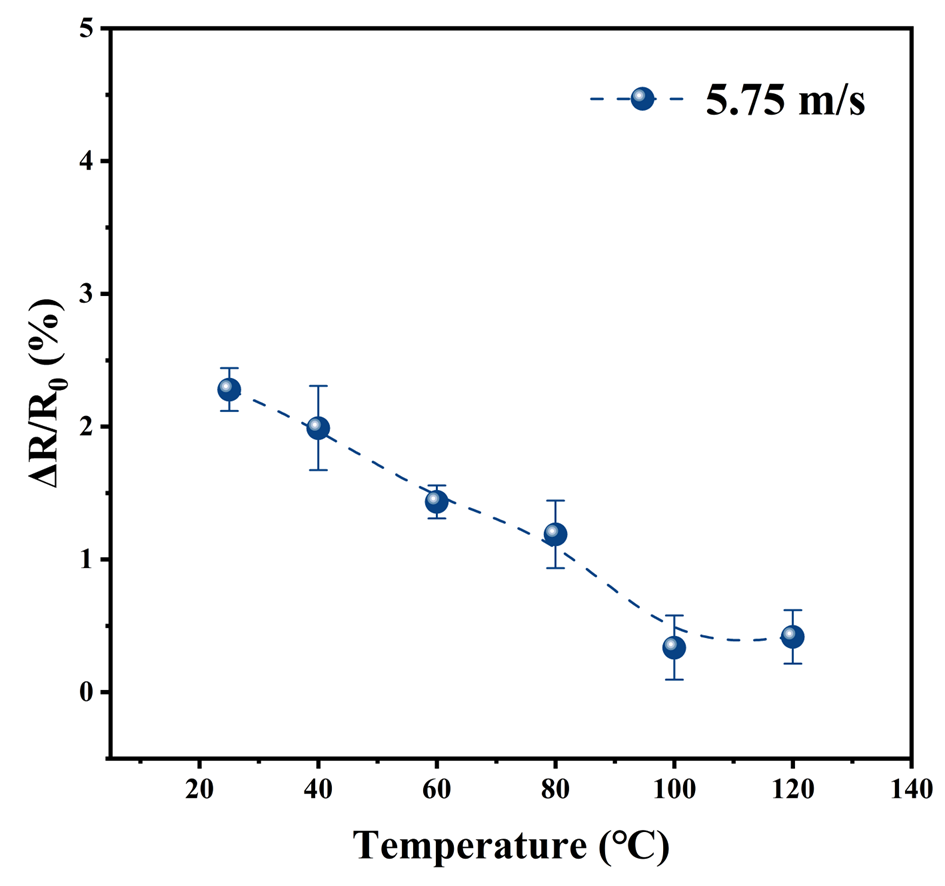


**Figure S12.** ΔR/R_0_ of the sensor for the same airflow velocity at different temperatures.


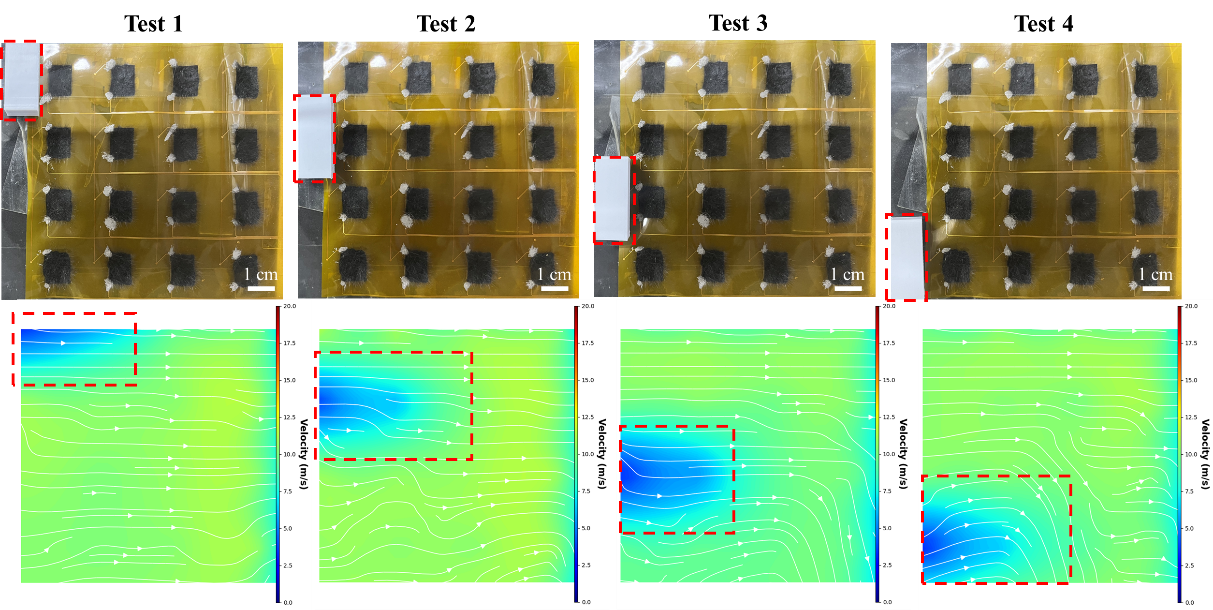


**Figure S13.** Test of local variations in airflow fields.

**Table S1.** Key parameters of the airflow sensors from the literature.

| **Record** | **Materials** | **Principle** | **Detection limit** | **Response time** | **Ref.** |
| --- | --- | --- | --- | --- | --- |
| 1 | Single silicon nanowire | Piezoresistive | 15.3 m s^-1^ | 0.04 s | 25 |
| 2 | Carbon nanotube | Piezoresistive | 7 m s^1^ | 1.3 s | 28 |
| 3 | Carbon fiber | Piezoresistive | 16 m s^1^ | 0.103 s | 32 |
| 4 | Carbon fiber | Piezoresistive | 2.66 m s^1^ | 1.7 s | 33 |
| 5 | LIG | Piezoresistive | 8 m s^1^ | 0.5 s | 23 |
| 6 | Carbon nanotubes | Piezoresistive | 10 m s^1^ | 0.1 s | 34 |
| 7 | Graphene/single-walled nanotubes-Ecoflex | Piezoresistive | 3 m s^1^ | 1.04 s | 26 |
| 8 | Carbon fiber/LIG | Piezoresistive | 18.36 m s^1^ | 0.03983 s | **This work** |

**Video S1.** Reponse of the fluff layer under airflow.

**Video S2.** Changes in carbon fiber separation and resistance in the fluffy layer under different airflow velocities.

**Video S3.** Visualization test comparing airflow field disturbances between the sensor in this work and commercial sensors.

**Video S4.** Continuous testing of the unsteady flow field of the sensor array.

**Video S5.** Applications test 1.

**Video S6.** Applications test 2.

**Video S7.** Applications test 3.
